# Diversity‐On: A Diversity‐Sensitive Online Self‐Help Program for Family Caregivers—A Protocol for a Mixed Methods Study

**DOI:** 10.1111/jan.16443

**Published:** 2024-09-10

**Authors:** Kübra Annac, Mualla Basyigit, Sümeyra Öztürk, Ela Rana Örs, Tugba Aksakal, Christina Kuhn, Anja Rutenkröger, Hürrem Tezcan‐Güntekin, Yüce Yilmaz‐Aslan, Patrick Brzoska

**Affiliations:** ^1^ Faculty of Health/School of Medicine, Health Services Research Unit Witten/Herdecke University Witten Germany; ^2^ Berlin School of Public Health Alice Salomon University Berlin Berlin Germany; ^3^ Demenz Support Stuttgart gGmbH Stuttgart Germany

**Keywords:** caring relatives, dementia, diversity, mixed methods, online, self‐help, study protocol, support group

## Abstract

**Background:**

Scientific research has consistently emphasised the high levels of stress encountered by family caregivers of individuals living with dementia. However, conventional self‐help approaches remain underutilised. The ‘Diversity‐On’ study addresses this issue. The study employs a storytelling approach to develop and evaluate an online self‐help program that is participatory and diversity‐sensitive, thereby ensuring congruence with diverse identities and lifeworlds.

**Methods:**

The study uses a mixed‐methods design, comprising the allocation and implementation of the intervention, the development of stories, a process evaluation (*N* = 20) and an outcome evaluation (quantitative: *N* = 130, qualitative: *N* = 20). The study's primary focus is its comprehensive participatory approach, integrated throughout the research process. The study is dependent on the input of a number of stakeholders, all of whom are committed to advocating for the concerns of patients.

**Discussion:**

Given its participatory methodology and intersectional perspective, the ‘Diversity‐On’ study is anticipated to yield a number of significant outcomes. The study has the potential to empower family caregivers of individuals living with dementia who are under high stress, empowering them to take part in self‐help groups despite multiple barriers, thus alleviating their burden. Additionally, it has the capacity to promote the well‐being of caregiving relatives who are providing care and are experiencing high levels of stress. The study's objective is to maintain home care arrangements for as long as possible, in accordance with the values and preferences of care recipients and their families. The study intends to develop and assess a customised online self‐help resource that is suitable for a diverse range of users and that remains accessible beyond the study period.

**Trial Registration:**

The project is subsequently registered in ClinicalTrials.gov

**Summary**

- This is a protocol for a public health intervention, particularly in the area of nursing care, that aims to improve the health outcomes of the population, with a particular focus on vulnerable groups.
- The study can help to improve home care, maintain the setting and relieve the burden on the healthcare sector.
- The present protocol contains a detailed description of the hypotheses, rationale and methodology of the study to ensure transparency, reduce publication bias and enhance the reproducibility of study design and analysis.

## 1 Introduction

As the number of individuals with dementia living at home is projected to rise worldwide, it is anticipated that there will be an increase in demand for home‐based care services as well (Prince et al. 2016). Family caregivers are expected to be the primary stakeholders providing essential support to their relatives (Ceci, Symonds Brown, and Purkis 2019). In Germany, the country in which this study will be conducted, 70% of individuals requiring care are cared for at home, with family members and relatives being the primary caregivers. Only 24.3% opt for outpatient care (Statistisches Bundesamt 2018). This proportion is lower than in many other industrialised nations, for example, Canada, where around half of the individuals living with dementia reside at home while receiving family support (Manuel et al. 2016). Particularly among individuals of Turkish origin in Germany, the percentage of those receiving home‐based care is considerably higher, at 98% (Okken, Spallek, and Razum 2008). As the number of individuals residing at home increases, so too will the demand for home‐based care. It is therefore evident that family caregivers will continue to play a significant role in maintaining the sustainability of the health and social care systems (Dawson et al. 2015).

For family caregiver providing care can often be physically and psychologically demanding (Kimura et al. 2019; Seidel and Thyrian 2019). In Germany, 48.7% of individuals involved in the provision of care for family members experience mental disorders, underscoring the considerable burden and subsequent consequences associated with caregiving responsibilities (Rothgang and Müller 2018). Mental illnesses represent the fourth leading cause of reduced healthy life years in Germany (Rabe‐Menssen, Dazer, and Maaß 2020), contributing significantly to the overall disease burden.

It is thus imperative that suitable assistance be made available to family caregivers in order to minimise the risks to their health, to prevent premature institutionalisation or substandard care for individuals with dementia and to sustain the effective functioning of health and social care systems (Schoenmakers, Buntinx, and DeLepeleire 2010). In order to support continuity of home care, it is essential that the primary caregivers have access to the necessary resources and that a properly functioning support network is in place. By enhancing the available resources for family caregivers, their individual coping strategies can be improved, leading to a substantial positive impact on their well‐being (Brügger, Jaquier, and Sottas 2016). In order to promote and protect the health of family caregivers, there is a need to focus on the cultivation of established coping mechanisms. A particular emphasis must be placed on the development of self‐management skills among family caregivers, with the objective of effectively mitigating the impact of various stressors (Oliveira, Sousa, and Orrell 2019; Huis In Het Veld et al. 2016). In light of the pivotal role of self‐management in the prevention of chronic diseases, it is evident that the empowerment of family caregivers is of paramount importance (Toms et al. 2015). Given that family caregivers are more susceptible to stress‐related chronic illnesses, the implementation of user‐oriented preventive measures is crucial to prevent diseases associated with overwork and burnout.

Support services such as traditional self‐help formats see limited uptake among specific population groups in Germany, including migrants (Kofahl et al. 2019). Consequently, the highly heterogeneous migrant population is particularly vulnerable in this context, as, for example, the proportion of people who are cared for at home is as high as 98% among people of Turkish origin in Germany. Qualitative studies conducted among the migrant community in German‐speaking countries, focusing on caregivers of individuals living with dementia, reveal that those caregivers choosing not to utilise support services or self‐help options can themselves become entangled in the ailment due to prolonged stress (Piechotta and Matter 2008). Research highlights that distancing themselves from their social environment can result in a distressing sense of isolation among family caregivers. Moreover, the fear of being stigmatised due to their caregiving responsibilities can further impede effective coping with both caregiving challenges and daily issues (Toms et al. 2015).

The risk of isolation and co‐illness could be mitigated through preventive and tailored interventions. These interventions should focus on addressing the caregivers' specific needs, enhancing their self‐management capabilities and empowering them through hands‐on support. By doing so, caregivers can regain a sense of control and autonomy. Directing one's attention away from stressful emotions, affording chances for relaxation and engaging with peers in comparable situations has the potential to alleviate the impact of responsibilities associated with caring for a loved one at home (Huis In Het Veld et al. 2016).

Currently, to the best of the authors' knowledge, no online self‐help platforms effectively cater to the diverse needs of migrants in Germany, despite the high prevalence of family caregivers within this population group who experience a high burden (Tezcan‐Güntekin and Razum 2018). Participants in a support group show considerable diversity in terms of age, life experiences, relationships with care recipients, religious beliefs, language proficiency, sexual orientation, educational attainment and the challenges associated with caring responsibilities. This heterogeneity has the potential to decrease the level to which individuals identify with their fellow group members, resulting in sporadic participation or even withdrawal from the group if differences are not appropriately addressed (Yılmaz‐Aslan et al. 2021). Consequently, online self‐help communities that are sensitive to this diversity must be established. Such an approach holds the potential to foster a substantial sense of belonging and continuity among participants, thereby maximising involvement within these groups.

Storytelling could be a beneficial strategy in self‐help groups (Greenhalgh 2017; Lewis 2011). This narrative approach holds significant potential for advancing health literacy, as well as bolstering self‐efficacy expectations and improving self‐management skills, particularly for family caregivers (Holm, Lepp, and Ringsberg 2005; Glodny, Yilmaz‐Aslan, and Razum 2011). The method entails arranging storytelling sessions that cover a wide variety of subjects, with the principal objective of enabling individuals to exchange information, personal encounters and perceptions (Glodny, Yilmaz‐Aslan, and Razum 2011). In a previous study by one of the co‐authors of the present paper (YYA), storytelling was implemented in the context of home care and was directed at Turkish immigrants. Within the study, a self‐help‐centred storytelling intervention was developed to provide family caregivers with a platform to freely discuss their caregiving circumstances and challenges they faced while sharing care‐related information. The methodology of generating narratives resulted in a significant exchange of knowledge, experiences and insights among the family caregivers involved. This highlighted techniques to minimise barriers to accessing support services and to maximise the unused potential for self‐help among family caregivers, while emphasising the fundamental importance of engaging the target audience in the design of such services (Kofahl et al. 2019).

As previously stated, self‐help formats are infrequently used by family caregivers of patients living with dementia. The objective of the ‘Diversity‐On’ study is to create an online self‐help platform that promotes diversity, encourages participation and takes into account the diversity of participants. This self‐help platform will establish a guided group specifically for caregivers of individuals living with dementia, utilising the previously mentioned storytelling methodology. The platform will be implemented for people with a Turkish migration background. This group is intended to serve as an example of a wide range of heterogeneous and diverse people who require individualised and diversity‐sensitive support, while also considering the intersectionality with other diversity characteristics. The online self‐help platform is designed to connect family caregivers who share common diversity characteristics or are in similar care situations, with a focus on enhancing their self‐efficacy. By doing so, it aims to alleviate the burden associated with their caregiving circumstances and expand their autonomy within their specific life contexts, ultimately strengthening their self‐management abilities.

Accordingly, the core research question of the ‘Diversity‐On’ study is: To what extent can a participatory and diversity‐sensitive online self‐help service, based on the storytelling approach, increase the self‐efficacy and reduce the burdens of caregiving relatives with a migration background, taking into account intersectional interrelationships with other diversity characteristics?

An outcome evaluation will determine the overall impact of the online self‐help service on self‐efficacy expectations and other related outcomes. As such, the study will examine the following primary and secondary hypotheses:

### 1.1 Main (Primary) Hypothesis

The online self‐help service can increase the self‐efficacy expectation of family caregivers with Turkish migration background by 2.5 units (assessed with the SWE questionnaire; see Section 2).

### 1.2 Secondary Hypotheses a–e

The online self‐help service can

- increase dementia‐related health literacy,
- reduce perceived stress,
- improve health‐related quality of life,
- enhance empowerment,
- lessen perceived burden

of family caregivers with a Turkish migration background.

### 1.3 Qualitative Hypothesis

The online self‐help service is presumed to have high usability.

### 1.4 Trial Design

The study uses a mixed‐methods design, consisting of the allocation and implementation of the intervention, the development of stories, process evaluation (*N* = 20) and outcome evaluation (quantitative *N* = 130, qualitative: *N* = 20).

## 2 Methods

This present protocol is reported in accordance with the reporting guidance provided by the SPIRIT checklist (Chan et al. 2013) (see Appendix 1), as recommended by the EQUATOR Network.

### 2.1 Participants, Interventions and Outcomes

#### 2.1.1 Study Setting

The study setting comprises the home care arrangements of individuals with dementia within the family unit. The principal objective of the study is to examine the processes involved in maintaining home care. The data will be collected in Germany, where the intervention will also be piloted and evaluated. It is reasonable to assume that the results can be applied internationally, and that the intervention can be applied in home care as well as in other care settings.

#### 2.1.2 Eligibility Criteria

##### 2.1.2.1 Study Participants

The objective of the intervention is to provide support to individuals of Turkish origin in Germany who are involved in the care of relatives living with dementia. The study will include all adults (aged ≥18 years) of Turkish origin who are responsible for the care of a relative living with dementia. Individuals who are not of Turkish origin, who are under the age of 18, or who are not responsible for the care of a relative living with dementia will be excluded from the study.

##### 2.1.2.2 Council of Family Caregivers

The online self‐help program will be guided and facilitated by a council of family caregivers that will actively participate in the study. Comprising 10–12 family caregivers, the council will represent a diverse range across gender, class, race and body (Winker and Degele 2011). The council members will collaborate with the project team in the secondary data analysis (see below) and will also oversee the intervention. The study benefits from their participation by incorporating individual perspectives in the analysis and contributing to the participatory design of the research (von Unger 2014).

##### 2.1.2.3 Expert Advisory Board

An additional expert advisory board will be established, consisting of nursing scientists, migration researchers, experts for health promotion and prevention, experts for participatory research and experts from the Turkish dementia community. The board will undertake ongoing, biannual monitoring and reflection of each study phase and interim findings.

#### 2.1.3 Interventions

The ‘Diversity‐On’ intervention consists of an online self‐help platform that promotes diversity, encourages participation and caters to the diversity of the participants. This self‐help platform will facilitate a guided group specifically for caregivers of individuals living with dementia, utilising the previously mentioned storytelling methodology. The platform will be implemented for people with a Turkish migration background.

In total, 130 family caregivers will be allocated to 26 online support groups of about 5–6 persons each, comprising both intervention and control (as described below). The 26 groups will be randomised into an intervention and a control group, each comprising 13 groups.

[Intervention]: The individuals randomly assigned to the intervention group are divided into 6 groups of approximately 6 individuals, with each group representing specific diversity characteristics. In the intervention group, online self‐help sessions will be delivered using the previously mentioned diversity‐sensitive storytelling approach. The 6 intervention groups will convene on a monthly basis to discuss one story at each meeting. All participating co‐researchers of the intervention groups will receive prior training on the utilisation and management of the stories, as well as on the conduct of an online support group (opening, story presentation, discussion facilitation and closing).

[Control]: The individuals randomly assigned to the control group will also be randomly divided into 6 groups, comprising approximately 6 individuals each. The 6 control groups will convene on a monthly basis to conduct conventional support group sessions that are not storytelling‐based and that are not specifically tailored to the participants' diversity sensitivity.

The self‐help groups will be managed by members of the council of family caregivers. In order to ensure the ethical integrity of the study, all meetings of both the intervention groups and the control groups will be attended by one member of the project team.

#### 2.1.4 Outcomes

The primary outcome of the evaluation is the caregivers' self‐efficacy expectations (see Section 1.1). Secondary outcomes (see Section 1.2) include health literacy, perceived stress, health‐related quality of life, empowerment and caregiver burden. All questionnaires used for the assessment are available in German and Turkish (as well as English) and have been validated by previous research (see Section 2.3.3).

#### 2.1.5 Participant Timeline

**FIGURE 1:**
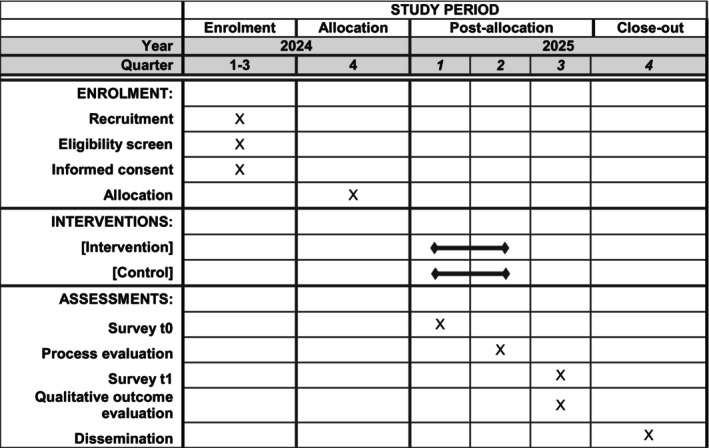
Time schedule of enrolment, interventions and assessments following the recommended SPIRIT template (Chan et al. 2013).

The Diversity‐On project is scheduled to run for a period of 36 months, starting in January 2023 and ending in December 2025. The intervention will be developed during the first year of the project. Recruitment of study participants will begin at the end of 2024. An overview of the timeline is given in Figure 1.

#### 2.1.6 Sample Size

Given a power of 80%, an alpha error of 5% and a standard deviation of 4 in the primary outcome measure and an estimated intraclass coefficient of 0.15, a sample size of a total of 26 clusters, each consisting of 5–6 caregivers, makes it feasible to identify mean differences of 2.5 on the SWE scale. This constitutes a medium effect size (Cohen's *d* = 0.6). The primary and secondary hypotheses will be assessed through multivariate testing using structural equation modelling.

#### 2.1.7 Recruitment

##### 2.1.7.1 Family Caregiver Recruitments for the Council

Recruiting family caregivers for the participatory council will be conducted together with voluntary welfare organisations and the project partners. Participation in the council is voluntary and requires informed consent. Participants in the council will receive written information about the study, the data protection concept and the declaration of consent in German and Turkish in advance. They will be given the opportunity to ask questions about the study before giving their written consent to participation.

##### 2.1.7.2 Participant Recruitments for the Online Self‐Help Groups

130 family caregivers are expected to take part in the evaluation (see Section 2.1.6). Assuming a dropout rate of about 17%, 156 family caregivers of people living with dementia will be recruited for the self‐help groups by means of various cooperation partners, including welfare and self‐help organisations and family doctors working in districts with a high proportion of migrants, as well as through social media (e.g., Facebook and Instagram). Participation in the self‐help groups, as well as participation in the council will be voluntary and will take place after written information has been provided and informed consent has been obtained.

### 2.2 Assignment of Interventions

#### 2.2.1 Allocation

130 family caregivers will be allocated to 26 online support groups of about 5–6 persons each (see Section 2.1.3). The 26 groups will then be randomised into an intervention and a control group, each comprising 13 groups.

#### 2.2.2 Implementation

The allocation sequence will be generated at random. The project team is responsible for enrolling participants and assigning them to either the intervention group or the control group. Subsequently, participants in both the intervention and control groups are allocated to small groups comprising 5–6 individuals. In the intervention group, the allocation will be made by the project team on the basis of the diversity characteristics of the participants. In the control group, the allocation process is randomised, with diversity characteristics not being taken into account.

### 2.3 Data Collection, Management and Analysis

#### 2.3.1 Development of Stories

32 qualitative interviews conducted by the authors of this article as part of other projects during 2018–2021 that involved family caregivers of individuals with Turkish heritage and a diagnosis of dementia (Tezcan‐Güntekin and Razum 2018; Tezcan‐Güntekin, Yilmaz‐Aslan, and Özer‐Erdoğdu 2020; Yilmaz‐Aslan et al. 2021) will be processed as secondary data. The secondary data analysis will focus on identifying thematic areas and relevant diversity characteristics, all of which are important for implementing online support groups that use storytelling. The interviews will be analysed in collaboration with the council of family caregivers using the qualitative multi‐level analytical approach by Winker and Degele (2011). Within this framework, diversity‐specific factors contributing to burden will be scrutinised through an intersectional lens across three dimensions: identities, structural factors and societal representations. The multi‐level analysis consists of 8 steps, from which not only relevant burdens can be determined, but which will also allow to identify which systemic and social challenges family caregivers are exposed to (Winker and Degele 2011).

The challenges in providing diverse care that were identified in the secondary data analysis will be developed into stories for the storytelling approach (Greenhalgh 2017). These stories will be tailored to the individual diversity characteristics of the participants and will serve as the foundation for the online support groups. Furthermore, the stories will be supplemented with relevant information and support materials that cater specifically to the identified diversity characteristics.

#### 2.3.2 Process Evaluation

Over the 6‐month period, a process evaluation will be conducted. It will comprise 20 problem‐centred interviews following the methodology outlined by Witzel (2000). In order to facilitate a systematic approach to identifying the underlying issues faced by individuals, the term ‘problem‐centred’ is employed as a central criterion within the methodology, encapsulating the notion of ‘problem‐centredness’. Situation‐specific, adaptable methodologies that facilitate the process of concretisation are essential. The core communication strategies of the problem‐centred interview encompass the initiation of discourse, general probing, targeted probing and ad hoc questioning (Witzel 2000). It is advisable that the subject matter be approached with an open mind and that the analysis process should aim for a more inductive generalisation. Accordingly, these interviews will be subjected to a summative qualitative content analysis in accordance with the methodology proposed by Mayring (2014). A sentence‐by‐sentence analysis will be used to ensure that statements about the content are made in context, that is, according to the stage of development of the conversation (Witzel 2000). The process evaluation will be used to examine the usability of the intervention as well as the impact of the stories shared among the groups. The subsequent outcome evaluation will use qualitative and quantitative measures.

#### 2.3.3 Outcome Evaluation

##### 2.3.3.1 Quantitative Outcome Evaluation

The intervention will be evaluated by means of a cluster‐randomised controlled design, consisting of the aforementioned 2 × 13 online support groups, each comprising 5–6 individuals.

The primary outcome of the evaluation is the caregivers' self‐efficacy expectation (see Section 1.1), assessed by means of the General Self‐Efficacy Expectancy scale (‘Skala zur Allgemeinen Selbstwirksamkeitserwartung’; SWE) (Lewis 2011). Prior research suggests that educational or supportive interventions such as the proposed story‐telling self‐help intervention may increase family caregivers' self‐efficacy by a minimum of 2.5 units on the SWE scale (Chan et al. 2013; Winker and Degele 2011).

Secondary outcomes (see Section 1.2) include health literacy, perceived stress, health‐related quality of life, empowerment and caregiver burden. Health literacy is assessed by means of the HLS‐EU‐Q questionnaire (von Unger 2014), a German‐language survey tool with 47 items for the assessment of health literacy in various target groups. Caregivers' perceived stress is assessed using the Perceived Stress Scale (PSS) (Tezcan‐Güntekin, Yilmaz‐Aslan, and Özer‐Erdoğdu 2020). Health‐related quality of life is measured using the SF‐12 questionnaire (Yilmaz‐Aslan et al. 2021). Empowerment will be assessed through a questionnaire developed in a previous study (Kofahl et al. 2019). Perceived burden is measured using the home care scale (HPS) (von Unger 2014). The questionnaires are available in German and Turkish, and participants will be able to choose the language of the questionnaires according to their own preferences.

##### 2.3.3.2 Qualitative Outcome Evaluation

A qualitative outcome evaluation will be conducted in addition to the quantitative evaluation. For this purpose, 20 problem‐centred interviews (Witzel 2000) (see Section 2.3.2) will be conducted with participants of the intervention group. The interviews will be analysed through summative qualitative content analysis according to Mayring (Mayring 2014) (see Section 2.3.2). Participants will be selected via theoretical sampling, taking into account aspects that emerge from the online self‐help groups.

The aforementioned research approach contributes towards an integrated evaluation through an explanatory mixed‐methods approach utilising a ‘convergence model’ as the triangulation design (Witzel 2000).

### 2.4 Ethics and Dissemination

#### 2.4.1 Research Ethics Approval

The study was approved by the Ethics Committee of Witten/Herdecke University (Ref. No. S‐50/2023). All participants are free to choose whether or not to take part in the surveys, the intervention and the council, and must provide informed consent to do so. Prior to providing written consent to participate in the study, all participants will receive written information about the study. All personal data will be stored and later destroyed in accordance with the data protection concept and ethical approval. Given the pseudonymous/anonymous nature of the survey, full disclosure, voluntary participation and the intention to publish anonymised data, no ethical concerns are associated with the study.

#### 2.4.2 Protocol Amendments

Notable changes to the established protocol will be disseminated through the registration process on ClinicalTrials.gov and through the planned publication of subsequent study findings.

#### 2.4.3 Consent or Assent

The participation of all individuals in the surveys, the intervention/control group and the family council is entirely voluntary and contingent on prior informed consent. Those participating in the family council and the intervention will be provided with written information regarding the study, the data protection policy and the informed consent form in German and Turkish in advance (see Appendices 2 and 3). Those wishing to participate in the study will be given the opportunity to ask questions about the study prior to providing their informed consent.

#### 2.4.4 Confidentiality

The information provided by study participants is treated in accordance with the highest standards of confidentiality and anonymity. All data protection requirements will be observed in accordance with the General Data Protection Regulation (GDPR). The online support group will be conducted via a data protection‐compliant online conferencing tool. The results of the study will be published in anonymised form. The findings of the study will be accessible to participants at any time. Participants will be given the possibility to withdraw from the study at any time without providing a reason and without incurring any disadvantages. It should be noted that data that has already been anonymised cannot be deleted or destroyed at the request of participants, as the process of anonymisation irreversibly removes the ability to attribute the data to a specific individual. The project management team will take all reasonable steps to ensure the protection of the study participants' data, in accordance with the GDPR and other relevant legislation. The data will be protected from unauthorised access. Should the study participants exhibit indications of self‐endangerment or harm to others, the ethics committee will be consulted regarding the subsequent course of action. The team adheres to the recommendations for safeguarding good scientific practice set out by the German Research Foundation (DFG), as well as the recommendations of Good Epidemiological Practice (Deutsche Gesellschaft für Epidemiologie [DGEpi] 2018) and the Declaration of Helsinki, as amended.

#### 2.4.5 Declaration of Interests

The principal investigators involved in the study have no financial or other competing interests at the study level or at the level of each study site.

#### 2.4.6 Access to Data

All personal data will be stored and later destroyed in accordance with the data protection policy. All documents will be stored securely on the premises of Witten/Herdecke University.

#### 2.4.7 Dissemination Policy

The objective of the validation of findings is to disseminate the results of the study in an extensive and systematic manner to audiences comprising the scientific, practical and public communities. The findings of the study will be disseminated to the academic community and relevant stakeholders through the publication of a final report and the organisation of a workshop. Additionally, the results will be presented at national and international conferences and published in relevant national and international journals.

From a pragmatic perspective, it is crucial to adopt a participatory approach to ensure the ongoing dissemination of both process and outcome evaluation findings throughout the duration of the study. The distribution of concise, single‐page information sheets to welfare organisations will reinforce the dissemination of information. Concurrently, the dissemination of information intended for the public will be enhanced by the distribution of multilingual project snippets designed for various social media platforms.

## 3 Discussion

It is not uncommon for family members who provide care for individuals with dementia to experience considerable stress. Traditional self‐help methods have only limited effectiveness. The ‘Diversity‐On’ project aims to develop and evaluate an online self‐help platform that is participatory and sensitive to diversity, ensuring the best possible match with various characteristics and lifeworlds.

An intersectional qualitative multi‐level analysis will be employed to develop care‐related open‐ended stories utilising the *storytelling* approach (Greenhalgh 2017). These narratives will be complemented by informative and supportive materials that address specific diversity characteristics, leading to the production of ‘storyboards’. At the beginning of self‐help meetings, stories will be jointly shared among participants, who will then discuss the presented scenarios and brainstorm potential strategies for addressing similar situations. This narrative‐driven approach establishes an environment where caregivers can openly discuss obstacles related to care provision.

The majority of current self‐help programs designed for family caregivers tend to neglect the diverse characteristics of their target population. ‘Diversity‐On’ employs an intersectional methodology to address the challenges faced by family caregivers. The objective is to develop a supportive program that is responsive to their unique lifeworlds and identity characteristics. The efficacy of self‐management skills and the utilisation of preventive measures and health promotion strategies among caregivers is contingent upon the presence of diverse individual attributes (Tezcan‐Güntekin and Razum 2018; Brand et al. 2015). Diverse characteristics interact with each other, a phenomenon that can be examined through intersectional analyses. This approach permits the examination of how multiple diversity attributes can intensify inequalities. An intersectional perspective is often limited in the domains of prevention, health promotion and nursing science. In particular, the interplay between migration and other diversity attributes has not been sufficiently investigated. It is postulated that the coexistence of multiple attributes, each of which may independently precipitate significant psychological distress and related health complications, may exacerbate the effects of stress and the risk of illness.

In this context, ‘Diversity‐On’ employs a strong *participatory approach*, which is integral to the entire research process. The study involves collaboration with various stakeholders who ardently advocate for patient interests. It employs a participatory research design (von Unger 2014) that (a) involves relevant group members in significant aspects of the research, (b) achieves fair collaboration between the study staff and co‐researchers (in this instance, family members), (c) engages and empowers family members as co‐researchers throughout the study period and (d) explores the assets of the affected communities in a resource‐focused way. The online self‐help service is developed in partnership with a council of family caregivers, promoting a co‐design approach that prioritises user orientation and resonance with diverse life contexts. These self‐help groups are guided by members of the council. Furthermore, council members collaborate with the project team to conduct secondary data analyses.

Nevertheless, the implementation of this participatory approach is not without its challenges. The recruitment of council members may prove to be a significant challenge, necessitating an accessible and undemanding approach. The research team has established connections within various networks and communities that facilitate access through gatekeepers, a strategy that has been supported by previous research. In light of the potential for disruptions due to caregiving responsibilities of participants during the participatory analysis of secondary qualitative data, it is essential to oversample and provide substantial assistance to the family members' council. It is important to acknowledge that some members may lack confidence in scientific work and, as a result, may choose to withdraw. It is crucial to recognise the potential for delays in the schedule due to the necessity of imparting fundamental research methodologies to council members. To enhance participant identification with the research as a whole, we advocate for active participant involvement. We provide an honorarium to reward contributions and encourage engagement, fostering a sense of belonging. Furthermore, participants serve as facilitators during self‐help group meetings, underscoring the significance of their participation and strengthening their identification with the study.

The diverse support group has the potential to effectively address the challenges faced by family caregivers. The act of exchanging experiences with individuals who are similarly situated serves to enhance a stronger sense of identification, which in turn fosters ongoing engagement. A sense of isolation is replaced by a perception of belonging. The provision of ongoing interactions has the potential to prevent the onset of mental health issues among family caregivers, as well as enhance their skills through the exchange of information within the group. It is anticipated that the acquisition of self‐management abilities will result in a reduction in stress and illness, addressing both the psychological and physical dimensions. This is likely to delay hospitalisation and the transfer of care recipients to institutional facilities. Collectively, the expected results of ‘Diversity‐On’ will assist highly stressed family caregivers of individuals with dementia in participating in self‐help initiatives by overcoming various obstacles, ultimately reducing their burden.

## Author Contributions

K.A. developed the initial draft of the manuscript. M.B. and S.Ö. were major contributors in writing the manuscript and together with K.A. are responsible for the project implementation and the processing of the work packages. E.R.Ö., T.A., C.K. and A.R. read and revised the manuscript. H.T.‐G. is responsible for project planning and implementation. Y.Y.‐A. and P.B. are responsible for project planning and design and corresponding work packages in the project. All authors read and approved the final manuscript.

## Ethics Statement

An ethics application was submitted to the Ethics Committee of the University Witten/Herdecke with application no. S‐50/2023. The ethics committee approved the study, and ethical and professional concerns were not raised. Participation in all surveys, the intervention and the council is voluntary and requires informed consent. All participants of the study receive written information about the study in advance before they give their written consent to participate. All personal data will be stored and destroyed in accordance with the data protection concept and the ethics approval.

## Conflicts of Interest

The authors declare no conflicts of interest.

### Peer Review

The peer review history for this article is available at https://www.webofscience.com/api/gateway/wos/peer‐review/10.1111/jan.16443.

## Supporting information


**Appendix 1:** Filled SPIRIT 2013 Checklist.

**Appendix 2:** Study information form given to study participants.

**Appendix 3:** Consent form given to study participants.

## Data Availability

The data that support the findings of this study are available on request from the corresponding author. The data are not publicly available due to privacy or ethical restrictions.
