# Supplementary material for: Diversity‐On: A Diversity‐Sensitive Online Self‐Help Program for Family Caregivers—A Protocol for a Mixed Methods Study
Source: J Adv Nurs. 2024 Sep 10;81(5):2810–8. doi: 10.1111/jan.16443 (PMC11967300; doi:10.1111/jan.16443)
Supplement: Supplementary file 2 — Appendix 2: Study information form given to study participants. [file JAN-81-2810-s001.docx]

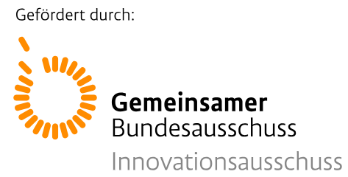

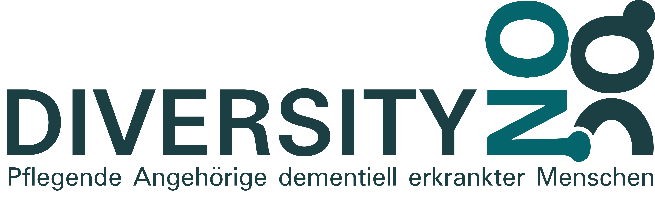


**Participant information**

**for the online self-help group in the Diversity-On project**

Ladies and gentlemen,

Family carers of people with dementia can experience a variety of stresses. Relief programmes such as traditional self-help formats do not always adequately meet their needs. An online self-help programme is being developed and evaluated as part of the Diversity-On study in order to find the best possible fit for different needs. The online self-help programme is a supported self-help group for family carers of people with dementia.

**Aim of the study:**

The aim of the study is to develop an online self-help programme to relieve the burden on family carers of people with dementia and to evaluate its effectiveness.

For this purpose, we would like to invite you to participate in the online self-help group. The online self-help programme is intended to enable participants to exchange ideas in an online self-help group with family carers with similar care situations and to network regardless of location, to relieve them according to their individual life and care situations, and to strengthen their health and self-management skills.

**Intervention process:**

The online self-help programme is a self-help group for family carers of people with dementia who are of Turkish origin. If you decide to take part in the self-help group, the project team will assign you to a group of around 6 people. The self-help group meetings will take place from October 2024 to March 2025 for a period of 6 months. The group will meet online once a month for one hour to exchange ideas. The group will be led by you and supported by us in the background. A project employee will act as your direct contact person for the entire duration of the programme. At the beginning and after 6 months, you will complete an online questionnaire to find out how you found the self-help group.

**Advantages and disadvantages for participants:**

Advantages: By participating, you are helping to investigate the feasibility and effectiveness of a programme designed to support family carers of people with dementia. We therefore very much hope that you will agree to participate in the online support group.

Disadvantages: Participation in the online self-help group involves a time commitment of 1 hour per month over a period of 6 months.

**Inclusion and exclusion criteria:**

Inclusion criteria: We include all adults (at least 18 years) who care for a family member of Turkish origin with dementia.

Exclusion criteria: People who do not fulfil the above conditions must unfortunately be excluded.

**Type of funding for the project:**

The Diversity-On project („Prävention und Gesundheitsförderung für pflegende Angehörige. Entwicklung und Evaluation eines diversitätssensiblen Online-Selbsthilfeangebots zur Stärkung des Selbstmanagements am Beispiel von Menschen mit türkischem Migrationshintergrund“) is a project funded by the Joint Federal Committee (Innovation Fund) and will run from 01/2023 to 12/2025.

**Notes on data protection:**

We will of course treat your details anonymously and in strict confidence. We fulfil all data protection requirements (in accordance with GDPR). The online self-help group is conducted using a data protection-compliant online conference tool. The study results are only published in anonymised form.

You can gain insight into the results of the study at any time. Please feel free to contact us in this regard (see contact details below).

1. The person responsible pursuant to Art. 4 para. 7 of the EU General Data Protection Regulation (GDPR) and project manager is: Prof. Dr Patrick Brzoska (Chair of Health Services Research, Faculty of Health / Department of Human Medicine, Witten/Herdecke University, Alfred-Herrhausen-Straße 50, 58448 Witten, Tel: 02302/926-78605, [patrick.brzoska@uni-wh.de](mailto:patrick.brzoska@uni-wh.de)).

You can reach the data protection officer of Witten/Herdecke University, Dipl.-Stat. Martin Rützler, at: Tel. 02302/926-722, e-mail: Martin.Ruetzler@uni-wh.de, Alfred-Herrhausen-Straße 50, 58448 Witten.

(2) You have the following rights towards the project management with regard to the data concerning you

- Right of access,
- Right to rectification,
- right to cancellation,
- Right to restriction of processing,
- Right to object to processing
- Right to data portability

insofar as these rights are not restricted by Section 27 (2) BDSG or other laws in the context of data processing for scientific purposes. You also have the right to complain to a data protection supervisory authority about the processing of your personal data. You can contact the State Commissioner for Data Protection and Freedom of Information of North Rhine-Westphalia at Tel. 0211/38424-0, e-mail: poststelle@ldi.nrw.de, Postfach 20 04 44, 40102 Düsseldorf.

If you have any concerns, questions or complaints about data processing and compliance with data protection requirements, you can of course also contact the project management at any time. You will find the contact details at the bottom of this document.

The project management will take all reasonable steps to ensure the protection of your data in accordance with the General Data Protection Regulation (GDPR) and other laws. The data is secured against unauthorised access.

To protect your health and safety, we reserve the right to consult with the ethics committee about further action if there is any indication that you or others may be at risk during the study.

1. You have the right to revoke your declaration of consent under data protection law at any time. The withdrawal of consent shall not affect the lawfulness of processing based on consent before its withdrawal.
2. The legal basis for data processing is Art. 6 para. 1 lit. a) and Art. 9 para. 2 lit. a) GDPR.
3. Reference to right of cancellation:

It is guaranteed that you can withdraw from the survey and cancel it at any time without giving reasons and without any disadvantage to you. Please note that data that has already been anonymised can no longer be deleted/destroyed on request, as it is no longer possible to identify you personally once the data has been anonymised.


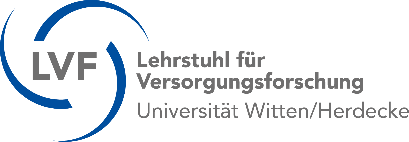
 **Contact:

Projekt management:**

### **
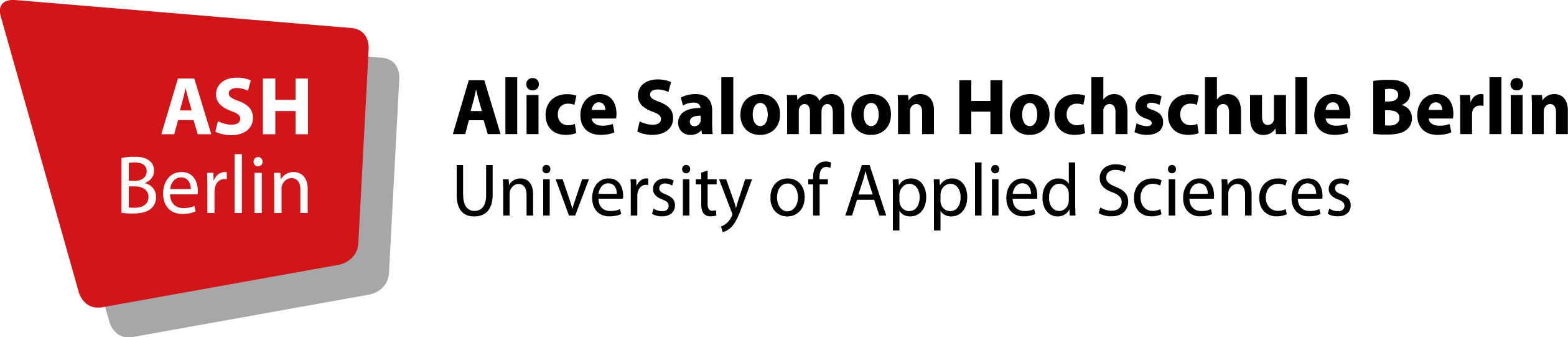
**


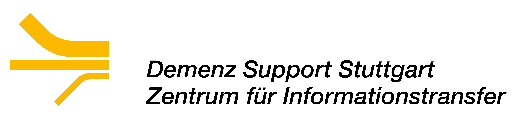
**Prof. Dr. Dr. Hürrem Tezcan-Güntekin** **Prof. Dr. Patrick Brzoska
Mualla Basyigit** **Dr. Yüce Yilmaz-Aslan**Alice Salomon University Berlin **Kübra Annac**
Alice-Salomon-Platz 5, 12627 Berlin Witten/Herdecke University
Tel.: 0157/54392724 Health Services Research Unit
E-Mail: [basyigit@ash-berlin.eu](mailto:basyigit@ash-berlin.eu) Alfred-Herrhausen-Str. 50, 58448 Witten Tel.: 02302/926-78674
 E-Mail: kuebra.annac@uni-wh.de

**Christina Kuhn
Dr. Anja Rutenkröger
Sümeyra Öztürk**
Demenz Support Stuttgart gGmbH
Zentrum für Informationstransfer
Zeppelinstraße 41, 73760 Ostfildern
Tel.: 0711/99787-15
E-Mail: s.oeztuerk@demenz-support.de

### **Prof. Dr. Patrick Brzoska**

### **Dr. Yüce Yilmaz-Aslan Kübra Annac** **Universität Witten/Herdecke** Lehrstuhl für Versorgungsforschung Fakultät für Gesundheit/Department für Humanmedizin Alfred-Herrhausen-Str. 50, 58448 Witten Tel.: 02302/926-78674

### E-Mail: kuebra.annac@uni-wh.de
