# Supplementary material for: Diversity‐On: A Diversity‐Sensitive Online Self‐Help Program for Family Caregivers—A Protocol for a Mixed Methods Study
Source: J Adv Nurs. 2024 Sep 10;81(5):2810–8. doi: 10.1111/jan.16443 (PMC11967300; doi:10.1111/jan.16443)
Supplement: Supplementary file 3 — Appendix 3: Consent form given to study participants. [file JAN-81-2810-s003.docx]

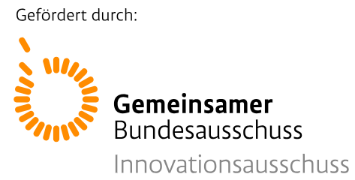

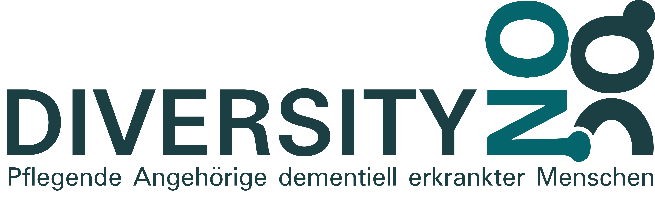


**Consent form for participation**

**in the online self-help group in the Diversity-On project**

I confirm that I have been informed about the objectives, procedure, duration and benefits of the project. I have read the participant information (or it was read to me) and I have understood it. In addition, I was also informed in a detailed and comprehensible manner by a project staff member about the purpose and procedure of the study, as well as about the benefits and risks that may be associated with the study.

In particular, I am aware of the extent to which, on what legal basis and for how long my data will be stored, as well as what rights I have vis-à-vis the controller (see below) with regard to the data I have provided.

The project manager and controller in accordance with Article 4(7) of the EU General Data Protection Regulation (GDPR) is Prof Dr Patrick Brzoska (Chair of Health Services Research, Faculty of Health/Department of Human Medicine, Witten/Herdecke University, Alfred-Herrhausen-Straße 50, 58448 Witten, Tel: 02302/926-78605, patrick.brzoska@uni-wh.de).

All my questions have been answered to my satisfaction. I received a copy of the participant information and the consent agreement. I have had sufficient time to think about my decision to participate and to decide freely. I am aware that participation is voluntary and that I can withdraw my consent at any time (verbally or in writing) without giving reasons and without any disadvantages for me. My data will then be completely deleted (unless it has already been anonymised). I will receive a message about this.

**By submitting the registration form, I declare that I am willing to participate voluntarily in the above-mentioned study and consent to the associated processing of my data, of which I am aware.**
